# Pan-immune-inflammation value is associated with the clinical stage of colorectal cancer

**DOI:** 10.3389/fsurg.2022.996844

**Published:** 2022-08-12

**Authors:** HanZheng Zhao, Xingyu Chen, WenHui Zhang, Die Cheng, Yanjie Lu, Cheng Wang, JunHu Li, LiuPing You, JiaYong Yu, WenLong Guo, YuHong Li, YueNan Huang

**Affiliations:** ^1^ Department of General Surgery, The Second Affiliated Hospital of Harbin Medical University, Harbin, China; ^2^ College of Bioinformatics Science and Technology, Harbin Medical University, Harbin, China; ^3^ Department of Pain Medicine, Harbin Medical University Cancer Hospital, Harbin, China; ^4^ Cancer Research Laboratory, Chengde Medical College, Chengde, China

**Keywords:** pan-immune-inflammation value, CEA, CA19-9, TNM stage, colorectal cancer

## Abstract

**Objective:**

We investigated the clinical significance of preoperative pan-immune-inflammation value (PIV) in patients with colorectal cancer (CRC).

**Methods:**

In this retrospective study, 366 cases who underwent surgery for CRC were enrolled. Their clinical data were collected. PIV was calculated with the formula PIV = [neutrophil count (10^9^/L)× platelet count (10^9^/L) × monocyte count (10^9^/L) /lymphocyte count (10^9^/L). Patients were divided into high PIV (> median PIV) and low PIV (< median PIV) groups. The relationship between PIV and clinicopathological features of CRC was investigated. Receiver operating characteristic (ROC) curve was plotted to indicate the value of immune-inflammatory biomarkers (IIBs) in predicting the TNM stage of CRC, and the area under the curve (AUC) was calculated to evaluate the actual clinical value of IIBs. AUC > 0.5 and closer to 1 indicated the better predictive efficacy. The influencing factors of PIV in CRC were analyzed.

**Results:**

We found that PIV was positively correlated with tumor size (*r* = 0.300, *p* < 0.05), carcinoembryonic antigen (CEA) (*r* = 0.214, *p* < 0.05) and carbohydrate antigen 125 (CA-125) (*r* = 0.249, *p* < 0.05), but negatively correlated with albumin (Alb) (*r* = −0.242, *p* < 0.05). PIV was significantly different in patients with different tumor locations (left or right), surgical methods (laparotomy versus laparoscopic surgery) (*p* < 0.05), and patients with different pathological T stages, N-stage and TNM stages (*p* < 0.05). ROC curve analysis of IIBs showed the AUC of PIV was greater than other markers when combined with CEA or carbohydrate antigen 19–9 (CA19–9). Multivariate regression analysis identified T stage, CEA, Alb, and tumor size as the independent influential factors of PIV in CRC.

**Conclusion:**

PIV is associated with the tumor stage in patients with CRC, which may be useful in preoperative assessment of CRC.

## Introduction

Colorectal cancer (CRC) is the third most common malignancy and its mortality rate ranks second in cancer-related mortality worldwide. The incidence of CRC in developed country is estimated to be 4-fold higher than that in developing country (1). In China, CRC is the third most common cancer, and its mortality rate ranks fifth. The CRC incidence in China continues to increase (2). Due to the lack of early symptoms, a considerable number of CRC patients are diagnosed at an advanced stage, with a poor outcomes (3). Thus, potential biomarkers should be investigated to improve early diagnosis and tumor staging.

As the basis for clinical staging and gold standard for predicting prognosis for CRC patients, TNM staging [local tumor spread (T stage), lymph node spread (N stage) and metastasis (M stage)] plays an important role in preoperative management, treatment selection, and postoperative management of CRC in the clinical practice. Studies have indicated that the TNM staging of CRC is affected by serum tumor markers (TMs) (4), prognostic nutritional index, systemic inflammatory response (5), and the immune microenvironment (6).

Common biomarkers of CRC, such as carcinoembryonic antigen (CEA) and carbohydrate antigen 19–9 (CA19–9) of TMs, have the merits of simplicity, availability, and robustness. They are generated and released during tumorigenesis, and can potentially reflect tumor changes at cellular levels (7, 8). However, TMs are mostly tumor-related, and host-related factors as markers are less reported. Recently, new evidence has shown that host-related factors such as inflammation status and immune response play an important role in the occurrence and development of tumor (9, 10). Immune-inflammatory biomarkers (IIBs) can reflect the balance between the status of inflammation and immunity in the host. IIBs such as neutrophil-to-lymphocyte ratio (NLR) and platelet-to-lymphocyte ratio (PLR) show prognostic values in CRC (11, 12), and systemic immune-inflammation index (SII) has also been shown as an effective predictor of CRC (13). Pan-immune-inflammation value (PIV), a new biomarker, has been introduced recently. PIV has been shown as a prognostic marker in HER-2 (+) breast cancer (14). High PIV (> median) was reported to be correlated with poor clinical outcomes in patients with small cell lung cancer (15). Fucà et al reported that, compared with SII, PIV was superior in evaluating progression-free survival and overall survival in patients with metastatic CRC (16).

In the present study, we investigated the association between PIV and clinicopathological features of CRC and compared the predicting values of NLR, SII, and PIV in TNM-stage of CRC patients who received radical surgery.

## Materials and methods

### Patients

Patients with primary CRC who received radical surgery at the Second Affiliated Hospital of Harbin Medical University between 2017 and 2022 were selected retrospectively. The inclusion criteria were: (a) patients had primary CRC confirmed by colonoscopy or postoperative histopathology; (b) patients underwent radical surgery; (c) patients were >18 years old; and (d) patients had complete and reliable clinical data, such as medical history, surgical records, and pathological results. The exclusion criteria were: (a) patients had history of other malignancies or were concomitant with other primary cancers; (b) patients had clinical evidence of any infection before surgery; (c) patients had hematological system diseases and autoimmune diseases; (d) patients had taken medications that might affect peripheral blood cells counts within 6 months; (e) patients were previously treated with neoadjuvant chemotherapy or radiotherapy; (f) patients underwent emergency surgery due to complications such as bowl obstruction or perforation. Finally, a total of 366 patients were included in this study. Preoperative laboratory data were retrieved from all patients. This study was approved by the Human Ethics Committee of the Second Affiliated Hospital of Harbin Medical University (KY2022–160). The informed consent was waived owing to the retrospective nature of this study. The study was conducted in accordance with the Declaration of Helsinki (revised in 2013).

### Data collection

The following variables were collected: sex, age, body mass index (BMI), albumin (Alb), CEA, CA19–9, CA-125, alpha-fetal protein (AFP), tumor location, tumor size, degree of tumor differentiation, surgical approach, duration of operation, T stage, N stage, M stage, and TNM stage. Numbers of lymphocytes, neutrophils, monocytes, and platelets in preoperative blood were retrieved. The NLR, SII, and PIV were calculated correspondingly: NLR = neutrophil count (10^9^/L)/lymphocyte count (10^9^/L); SII = [neutrophil count (10^9^/L)×platelet count (10^9^/L)]/lymphocyte count (10^9^/L); PIV = [neutrophil count (10^9^/L)  × platelet count (10^9^/L)  × monocyte count (10^9^/L)]/lymphocyte count (10^9^/L). CRC in the cecum, ascending colon, and transverse colon was defined as right-sided CRC. CRC in the descending colon and rectum was defined as left-sided CRC. Tumor staging was performed according to the 7th edition of the Union for International Cancer Control-American Joint Committee on cancer classification for CRC.

### Statistical analysis

Data were analyzed using the R language (version 3.5.1). Data with normal distribution were presented as mean ± standard deviation and compared with Student's t test. Qualitative data were presented as absolute numbers or percentages and *χ*² test was used for comparison. Data with skewed distribution were presented as median (P25, P75) and analyzed with non-parametric rank-sum test. Mann Whitney U non- parametric test was used for ranked data comparison. Variables were also analyzed with Pearson correlation coefficient. The ranked data were analyzed by Wilcoxon or Kruskal-Wallis test and corrected with Bonferroni correction. Receiver operating characteristic (ROC) curve analysis was performed and the area under the ROC curve (AUC) was calculated. A p value <0.05 was considered statistically significant.

## Results

### Patient characteristics

A total of 366 CRC patients, including 215 (59%) males and 151 (41%) females, were included in the present analysis (Table 1). Among them, 217 (59%) patients were older than 60 years. The numbers of patients with right-sided and left-sided CRC were 97 (27%) and 269 (73%), respectively. There were 104 (28%) patients with tumor size larger than 5 cm. Laparotomy was performed in 160 (44%) patients and laparoscopic surgery was performed in 202 (56%) patients. Additionally, 234 (64%) patients had stage I-II CRC, and 132 (36%) had stage III-V CRC. The median value of PIV was 159.95 (93.35, 256.17). Based on this, patients were divided into high PIV (PIV >159.95) and low PIV (PIV ≤159.95) groups.

**Table 1:** Patients’ characteristics.

Variables	*n*	%
Age (years)		
≤60	149	41
>60	217	59
Gender		
Female	151	41
Male	215	59
Localization		
Right	97	27
Left	269	73
Size		
≤5	262	72
>5	104	28
Surgical approacha		
Open	160	44
Laparoscopy	202	56
Pathological stage		
Stage I–II	234	64
Stage III–IV	132	36
PIV		
≤159	183	50
>159	183	50

^a^ Missing in 4 cases.

### Relationship between PIV and clinicopathological features in CRC patients

**Figure 1:**
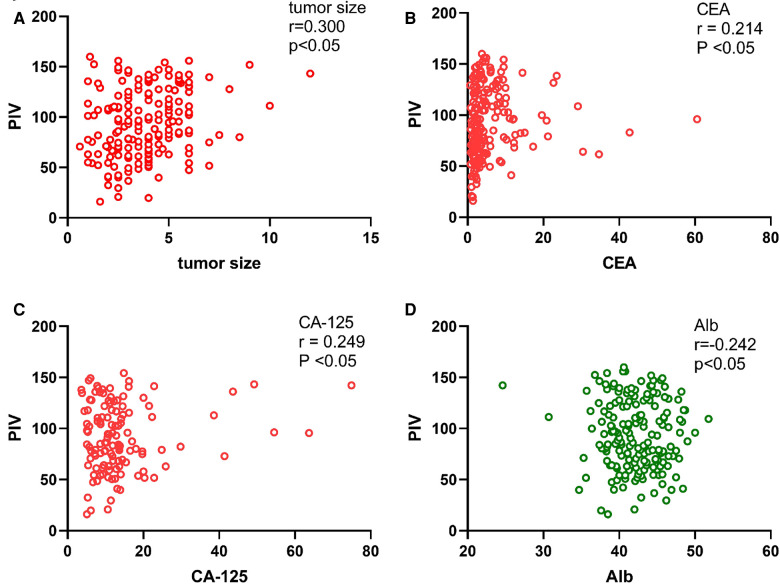
Correlation between PIV and tumor size, CEA, CA-125, Alb. Correlation between PIV and tumor size (**A**); Correlation between PIV and CEA (**B**); Correlation between PIV and CA-125 (**C**); Correlation between PIV and Alb (**D**).

There were statistically significant differences in Alb, CEA, tumor location, tumor size, surgical approach and pathological T-stage, N-stage and TNM-stage between patients with high PIV and low PIV (*p* < 0.05, Table 2). However, there were no differences in gender, age, CA-125, AFP, CA19–9, BMI, histological type, and pathological M-stage. In addition, cases in the high PIV group were more likely to have low Alb level, larger tumor size, and advanced T stage compared with those in the low PIV group. Patients whose tumors on the right side of the colon seemed to have higher PIV (Table 2). Pearson's correlation coefficient showed that PIV was positively correlated with tumor size (*r* = 0.300, *p* < 0.05), CEA (*r* = 0.214, *p* < 0.05), and CA-125 (*r* = 0.249, *p* < 0.05), but negatively correlated with Alb (*r* = −0.242, *p* < 0.05). However, PIV was not correlated with age, CA19–9, or AFP (*r* = 0.039, 0.096, and −0.062, respectively; *p* > 0.05) (Table 3A). In addition, we draw a scatter diagram to show the correlation between PIV and the above variables with statistical differences (Figures 1A–D). PIV in patients with left-sided CRC and right-sided CRC was 141.711 (86.210, 237.956) and 220.441 (106.635, 325.176), and in patients undergoing laparotomy and laparoscopic surgery was 188.319 (104.274, 283.652) and 138.109 (84.810, 242.166), respectively. PIV was significantly different in patients with different tumor locations and surgical approach (laparotomy vs laparoscopic surgery) (*p* < 0.05) (Table 3B). PIV of patients with pathological T-stages (T1, T2, T3, and T4 stage) was 135.645 (70.793, 208.382), 142.129 (95.878, 220.441), 166.963 (98.100, 283.329), and 270.964(207.017, 786.381), respectively. PIV of patients with pathological TNM-stages (I, II, III, and IV stage) was 139.409 (81.332, 214.575), 174.511 (95.970, 289.303), 152.257 (104.278, 263.991), and 203.870 (98.091, 302.717), respectively. There were statistically significant differences in PIV between patients with different pathological T stages and TNM stages (*p* < 0.05) (Table 3C). Then, we compared PIV of patients with different pathological T stages and TNM stages. When T1 stage was compared with T2, T3, T4, respectively, and when T2 was compared with T3 and T4 patients, the differences were statistically significant (*p* < 0.05). Pairwise comparison between other pathological T stages showed no significant differences (*p* > 0.05). There were statistically significant differences in PIV when TNMI stage was compared with II, III and IV stages, and when TNM II was compared with III (*p* < 0.05). Pairwise comparison of other pathological TNM stages showed no significant differences (*p* > 0.05) (Table 3D).

**Table 2:** Correlation between PIV and clinicopathological features in CRC patients.

Variables	Low PIV (*n* = 183)	High PIV (*n* = 183)	*p*-value
Age (year, $\bar{x}\pm S$)	62 ± 11	63 ± 11	0.4558
Gender			0.5955
Male	105	110	
Female	78	73	
BMI (kg/m^2^, $\bar{x}\pm s$)^a^	23.5 ± 4.1	23.9 ± 3.4	0.1643
Alb (g/L, $\bar{x}\pm s$)	42.3 ± 3.6	41.2 ± 4.3	0.01981
CEA	1.935 (3.300,6.580)	2.435 (4.680,10.255)	0.0006496
CA19-9	3.40 (7.05,15.92)	3.940 (9.140,20.615)	0.1278
CA125^b^	8.200 (11.300,19.275)	8.200 (11.200,18.800)	0.3022
AFP^c^	2.076 (2.660,3.763)	1.828 (2.545,3.445)	0.1316
Tumor Location			0.00645
Right	37	60	
Left	146	123	
Tumor Size	4.003 ± 1.791	5.026 ± 1.923	0.000000002151
Surgical Approach^d^			0.03429
Open	70	90	
Laparoscopy	111	91	
Operative Time^e^	177.547 ± 57.732	179.995 ± 59.988	0.6927
Histological Type			0.1823
Low	19	29	
Medium	157	147	
High	7	7	
T stage			0.00000000000005164
T1	25	3	
T2	70	11	
T3	86	158	
T4	2	11	
N stage			0.000002474
N0	150	102	
N1	23	53	
N2	10	28	
M stage			0.06237
M0	172	162	
M1	11	21	
TNM stage			0.0000000000006989
I	74	6	
II	71	83	
III	27	73	
IV	11	21	

^a^ Missing in 25 case.

^b^ Missing in 70 case.

^c^ Missing in 70 case.

^d^ Missing in 4 case.

^e^ Missing in 4 case.

**Table 3A:** Pearson's correlation coefficient.

Variables	*r*	*p*-value
Age	0.03957461	0.4504
Alb	−0.2416597	0.00001164
Tumor Size	0.3004814	0.00000003592
CEA	0.2148919	0.0000678
CA19-9	0.09573459	0.08977333
CA125^a^	0.2489981	0.00003893333
AFP^b^	−0.06226999	0.3264

^a^ Missing in 70 case.

^b^ Missing in 70 case.

**Table 3B:** Wilcoxon test.

	Variables	Distribution	*p*-value
Gender	Male	160.253 (99.531, 269.980)	0.1254
	Female	151.951 (80.287, 243.079)	
Tumor Location	Right	220.441 (106.635, 325.176)	0.00801
	Left	141.711 (86.211, 237.956)	
Surgical Approach^a^	Open	188.320 (104.274, 283.652)	0.02787
	Laparoscopy	138.109 (84.810, 242.166)	

^a^ Missing in 4 case.

**Table 3C:** Kruskal-Wallis test for T, N, M and TNM stage.

	Variables	Distribution	*p*-value
T stage	T1	135.645 (70.793, 208.382)	0.0000000000000022875
	T2	142.130 (95.879, 220.441)	
	T3	166.963 (98.100, 283.329)	
	T4	270.964 (207.017, 786.381)	
N stage	N0	132.556 (80.340, 225.310)	0.000000008983333
	N1	220.655 (137.205, 321.471)	
	N2	249.423 (160.381, 466.006)	
M stage	−	−	0.06837
TNM stage	I	139.410 (81.332, 214.575)	0.0000000000000011
	II	174.511 (95.970, 289.303)	
	III	152.257 (104.278, 263.992)	
	IV	203.870 (98.091, 302.718)	

**Table 3D:** Kruskal-Wallis test for different T stages and different TNM stages.

	Variables	*p*-value
T stage	T1/T2	0.04236
	T1/T3	0.0000000489
	T1/T4	0.00000378
	T2/T3	0.0000000002286
	T2/T4	0.00002055
	T3/T4	0.06634
TNM stage	I/II	0.000000001374
	I/III	0.00000000000000132
	I/IV	0.0000001648
	II/III	0.00007425
	II/IV	0.15504
	III/IV	0.2614

### Prediction value of TMs combined with IIBs for TNM staging in CRC

**Figure 2:**
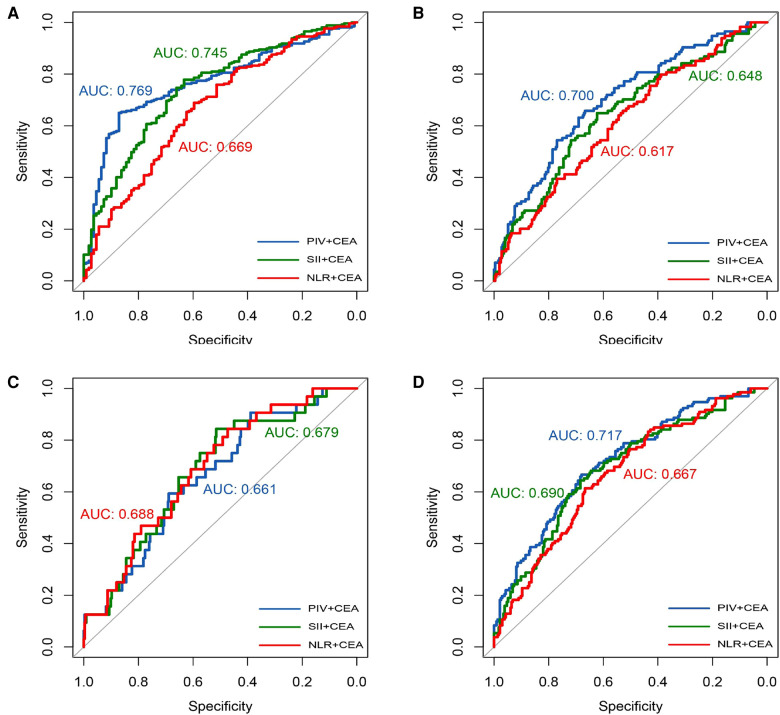
IIBS combined with CEA. Receiver operating curve analysis of T-stage (**A**); Receiver operating curve analysis of N-stage (**B**); Receiver operating curve analysis of M-stage (**C**); Receiver operating curve analysis of TNM-stage (**D**).

To more comprehensively reflect both the tumor characteristics and host systemic inflammatory status, we evaluated the prediction value of a combination of parameters (TMs and IIBs) for TNM staging in CRC. The AUCs of the NLR, SII, and PIV combined with CEA for pathological T-stage were 0.669, 0.745, and 0.769, respectively (Figure 2A); the AUCs for pathological N-stage were 0.617, 0.648, and 0.700, respectively (Figure 2B); the AUCs for pathological M-stage were 0.688, 0.679, and 0.661, respectively (Figure 2C); and the AUCs for pathological TNM-stage were 0.667, 0.690, and 0.717, respectively (Figure 2D).

**Figure 3:**
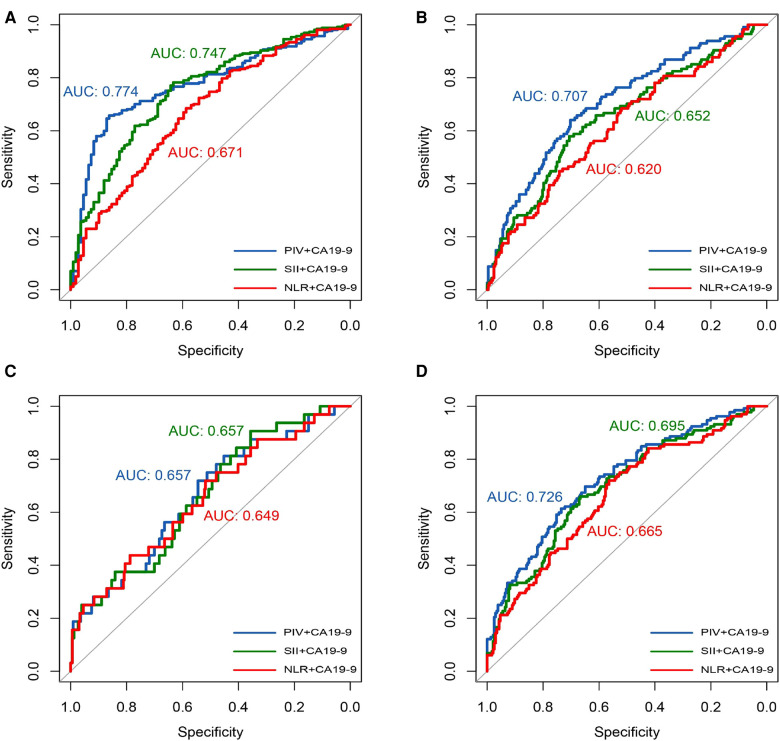
IIBS combined with CA19-9. Receiver operating curve analysis of T-stage (**A**); Receiver operating curve analysis of N-stage (**B**); Receiver operating curve analysis of M-stage (**C**); Receiver operating curve analysis of TNM-stage (**D**).

The AUCs of the NLR, SII, and PIV combined with CA19–9 for pathological T-stage were 0.671, 0.747, and 0.774, respectively (Figure 3A); the AUCs for pathological N-stage were 0.620, 0.652, and 0.707, respectively (Figure 3B); the AUCs for pathological M-stage were 0.649, 0.657, and 0.657, respectively (Figure 3C); and the AUCs for pathological TNM-stage were 0.665, 0.695, and 0.726, respectively (Figure 3D). Hence, among the IIBs analyzed in this study, PIV showed a superior prediction value for T, N, and TNM stages of CRC.

### Analysis of risk factors of PIV in CRC

To identify the factors that were linked with high PIV in CRC, we conducted univariate regression analysis. Totally, 15 variables were included in the univariate regression analysis. The results showed that BMI, Alb, CEA, CA-125, tumor size, tumor location, histological type, T-stage, N-stage, M-stage and TNM-stage were significantly linked with PIV (*p* < 0.05) (Table 4A). Then, we performed multivariate regression analysis. The influencing factors identified in univariate regression analysis and some factors that had great effects on PIV in clinical practice were included in the multivariate regression analysis. We found that CEA, CA-125, Alb, tumor size, and T stage were identified as the independent risk factors of PIV (Table 4B).

**Table 4A:** Univariate analysis of PIV in patients with CRC.

Variables	N	Beta	95% CI	*p-*value
Age	366	0.7568	−1.206–2.720	0.45036
Gender				
Female	151	1	−	−
Male	215	22.49	−22.899–67.883	0.332
BMI (kg/m^2^, $\bar{x}\pm s$)^a^	341	−7.098	−13.238–0.958	0.0241
Alb	366	−13.21	−18.656–7.759	0.00000291
CEA	366	0.8354	0.4453–1.225	0.0000339
CA19-9	366	0.16014	−0.011–0.331	0.0673
CA125^b^	296	2.175	1.208–3.142	0.0000146
AFP^c^	296	−10.421	−29.513–8.671	0.286
Localization				
L	269	1	−	−
R	97	83.06	33.085–133.031	0.00123
Tumor Size	366	33.622	22.658–44.586	0.00000000449
Histological type				
Low	48	1	−	−
Medium	304	−88.68	−154.580–22.778	0.00871
High	14	−118.71	−247.592–10.169	0.07185
T-stage				
T1	28	1	−	−
T2	81	40.94	−48.326–130.198	0.3693
T3	244	163.48	82.238–244.724	0.0000963
T4	13	294.25	157.599–430.904	0.0000309
N-stage				
N0	252	1	−	−
N1	76	141.72	88.379–195.052	0.000000321
N2	38	153.14	82.215–224.067	0.0000294
M-stage				
M0	334	1	−	−
M1	32	92.86	14.222–171.493	0.0212
TNM-stage				
I	80	1	−	−
II	154	103.43	48.831–158.031	0.000237
III	100	226.4	166.971–285.825	0.000000000000619
IV	32	208.33	125.464–291.198	0.00000127

^a^ Missing in 25 case.

^b^ Missing in 70 case.

^c^ Missing in 70 case.

**Table 4B:** Multivariate analysis of PIV in patients with CRC.

Variables	Beta	95%CI	*p*-value
BMI (kg/m^2^, $\bar{x}\pm s$)^a^	−1.5146	−7.909–4.879	0.64286
Alb	−6.0835	−11.374–0.793	0.024835
Tumor Size	19.5832	7.472–31.694	0.001662
Tumor Location			
Left	1	−	−
Right	41.8463	−3.663–87.355	0.072365
CEA	0.7009	0.340–1.061	0.000164
CA-125	1.3451	0.287–2.403	0.01332
Histological type			
Low	1	−	−
Medium	−25.5501	−100.970–49.870	0.50731
High	−1.4928	−167.649–164.663	0.98596
T-stage			
T1	1	−	−
T2	−22.6498	−109.702–64.403	0.610403
T3	113.6298	−7.354–234.614	0.066487
T4	173.9549	12.326–335.583	0.03561
N-stage			
N0	1	−	−
N1	60.6297	−46.635–167.895	0.268688
N2	17.3333	−103.814–138.481	0.779317
TNM-stage			
I	1	−	−
II	−86.1616	−184.690–12.367	0.087415
III	38.2899	−93.719–170.299	0.570059
IV	−9.6966	−135.886–116.493	0.880372

^a^ Missing in 25 case.

## Discussion

The interaction between systemic inflammation and local immune response plays an important role in the initiation, development, and progression of CRC (17, 18). IIBs such as NLR, PLR and SII have positive correlations with the poor outcomes of CRC patients (19, 20). IIBs involved in present study were based on peripheral blood cells. It has been reported that neutorphils act as a key factor in regulating tumor microenvironment (21). Tumor associated neutrophils have two different phenotypes (N1 and N2), owing to the effect of immunosuppression in tumor microenvironment. Tumor associated neutrophils mainly exist as N2 type. N2 type can promote the progression of tumors by expressing vascular endothelial growth factor and a variety of chemokines (CCL2, CCL5, etc.), can inhibit the anti-tumor activity of other immune cells, and can form cell clusters with circulating tumor cells (22–24). Activated platelets can secrete a variety of pro-inflammatory factors that attract circulating white blood cells to the sites of inflammation (25). It has been shown that circulating tumor cells can avoid the cytotoxicity of NK cells through expressing platelet-associated receptors or binding to platelets (26), thus promoting tumor cell metastasis. Tumor infiltrating lymphocytes are derived from lymphocytes in tumors, which play an important role in tumor microenvironment. They can mediate anti-tumor immune response *via* recognizing and killing tumor cells (27). High NLR and SII may indicate suppressed immune response in the body, which may alter tumor microenvironment and favor cancer initiation, progression, and metastasis. Recently, studies have shown that preoperative elevation of the peripheral absolute monocyte count is associated with prognosis of CRC patients (28). Under external stimulation, monocytes in peripheral blood are recruited into tumor microenvironment and activated as tumor associated macrophages. As the most abundant immune cells in the tumor microenvironment, tumor associated macrophages can promote the proliferation, invasion, migration and angiogenesis of tumor cells by secreting a variety of cytokines. In addition, tumor associated macrophages can also suppress the anti-tumor immune response of T cells by secreting chemokines, leading to an immunosuppressive state (29, 30).

In view of the important role of monocytes in promoting tumor progression, we tested PIV, which is a new type of IIBs based on peripheral neutrophil, platelet, monocyte and lymphocyte counts. The present study revealed interesting correlation between PIV and clinicopathological features in CRC patients. Hypoalbuminemia is a risk factor for poor prognosis in cancer patients (31). In this study, we found that PIV was positively correlated with tumor size and negatively correlated with Alb. This suggests that the patients with high PIV may be in a pro-tumor inflammation state, due to the bigger size of cancer and hypoalbuminemia. Patients with high PIV were more likely to have advanced T-stage compared with those with low PIV, implying a potential predictive value of PIV for T-stage. Tumorigenesis depends on the imbalance of the tumor promoting effects and the anti-tumor responses. Hence, separately using TMs or IIBs has certain limitations, and it is necessary to combine these biomarkers in further investigation.

Herein, we also evaluated the predictive ability of IIBs combined with CEA and CA19–9 for pathological T, N, and M stages in CRC patients. Interestingly, we found that the combination had potential in predicting TNM-stage, which further validates the above hypothesis that compared with other IIBs, PIV is a more comprehensive biomarker for assessing systemic inflammation. Thus, we assume that PIV can facilitate the assessment of inflammation/immune status in CRC patients preoperatively, and guide clinical treatment, which is worthy of further investigation.

Finally, we analyzed the risk factors of PIV. The results showed that the TNM-stage and tumor histological differentiation were the independent risk factors for PIV in CRC patients. We believe that this result emphasizes the distinctive characteristics of PIV in patients with CRC. Early clinical evaluation combined with PIV detection may be helpful to better evaluate the immune status of the body in the early stage of tumorigenesis, which may further guide clinical treatment.

The present study has some limitations. First, this study was a retrospective, single-center study. Therefore, a prospective validation study is needed to validate the results of the present study. Second, the sample size was relatively small. Third, only the patients who received radical surgery were enrolled, thus the results of the present study are not applicable for CRC patients whose tumors could not be surgically treated.

In conclusion, our study identifies PIV as a new type of IIBs strongly associated with the Alb, CEA, tumor location, tumor size, surgical approach, T-stage, N-stage, and TNM-stage of CRC patients. In addition, PIV has a predicting value for TNM-stage of CRC patients, and when compared with other IIBs, PIV is more capable in predicting T-stage, N-stage and TNM-stage. This study imply a clinical application value of PIV in evaluating inflammation/immune status in CRC patients and choosing treatment options preoperatively, which is worthy of further investigation.

## Data Availability

The original contributions presented in the study are included in the article/Supplementary Material, further inquiries can be directed to the corresponding author/s.
